# Mechanisms Linking Recurrent Bacterial Urinary Tract Infections to Chronic Kidney Disease Progression

**DOI:** 10.3390/ijms27114999

**Published:** 2026-05-31

**Authors:** Mariana-Emilia Caragea, Daniel Cosmin Caragea, Mohamed-Zakaria Assani, Isabela Siloși, Mihail Virgil Boldeanu, Lucrețiu Radu, Lidia Boldeanu, Cristin Constantin Vere

**Affiliations:** 1 Doctoral School, University of Medicine and Pharmacy of Craiova, 200349 Craiova, Romania; mariana.emilia77@yahoo.com (M.-E.C.); mohamed.assani@umfcv.ro (M.-Z.A.); 2 Department of Nephrology, Faculty of Medicine, University of Medicine and Pharmacy of Craiova, 200349 Craiova, Romania; daniel.caragea@umfcv.ro; 3 Department of Immunology, Faculty of Medicine, University of Medicine and Pharmacy of Craiova, 200349 Craiova, Romania; isabela_silosi@yahoo.com (I.S.); mihail.boldeanu@umfcv.ro (M.V.B.); 4 Department of Hygiene, Faculty of Medicine, University of Medicine and Pharmacy of Craiova, 200349 Craiova, Romania; 5 Department of Microbiology, Faculty of Medicine, University of Medicine and Pharmacy of Craiova, 200349 Craiova, Romania; 6 Department of Gastroenterology, University of Medicine and Pharmacy of Craiova, 200349 Craiova, Romania; vere_cristin@yahoo.com

**Keywords:** urinary tract infections, chronic kidney disease, uropathogenic *Escherichia coli*, *Klebsiella pneumoniae*, enterococcus, biomarkers, NGAL, inflammasome, oxidative stress, fibrosis, antibiotic resistance, microbiome

## Abstract

Urinary tract infections (UTIs) are among the most common bacterial infections worldwide and are traditionally considered acute and self-limited conditions. However, growing evidence suggests that recurrent or persistent UTIs may contribute to chronic kidney disease (CKD) progression through complex interactions between uropathogens and host responses. This review examines the pathophysiological links of UTIs caused by uropathogenic *Escherichia coli*, *Klebsiella* spp., and *Enterococcus* spp. and the development of chronic renal injury. Pathogen-specific persistence mechanisms, including intracellular survival, biofilm formation, and chronic colonization, may promote sustained inflammation, oxidative stress, and maladaptive repair responses. These processes are associated with tubular injury and progressive fibrotic remodeling. In addition, host-related factors such as diabetes, immune dysfunction, and antimicrobial resistance may further influence disease progression. Emerging biomarkers of inflammation, tubular injury, and fibrosis may improve early detection and risk stratification in patients with recurrent or complicated UTIs. Collectively, these findings support the concept that recurrent UTIs may represent potential contributors to CKD progression in susceptible individuals and highlight the importance of early recognition, pathogen-oriented management, and improved diagnostic strategies.

## 1. Introduction

The kidney serves not only as a filtration and metabolic organ but also as a site vulnerable to infectious insults that may contribute to progressive injury and remodeling. While classical risk factors such as hypertension, diabetes mellitus, and glomerular diseases dominate the pathogenesis of chronic kidney disease (CKD), accumulating evidence suggests that recurrent or severe urinary tract infections (UTIs), particularly recurrent pyelonephritis and complicated upper urinary tract infections, may represent underrecognized contributors to long-term renal decline [1,2]. By contrast, the association between uncomplicated lower UTIs and CKD progression remains less clearly established. Uropathogens such as *Escherichia coli* (particularly uropathogenic *E. coli*, UPEC), *Klebsiella* spp., and *Enterococcus* spp. are among the most frequent etiologic agents of UTIs and pyelonephritis, with distinct virulence traits that permit not only urinary colonization but also deeper tissue invasion and persistence [3,4,5].

These pathogens employ a repertoire of determinants, adhesins, fimbrial structures, toxins, iron-acquisition systems, and biofilm-forming capacity that facilitate ascending infection from the bladder to the upper urinary tract, evade host defenses, and confer resistance to antimicrobial therapy [6,7]. In experimental models, repeated renal infection induces local inflammation, tubular injury, and focal scarring, processes that may remain subclinical yet cumulatively predispose to progressive loss of function [8,9,10]. Mechanistically, stimulation of innate immune pattern-recognition receptors (e.g., Toll-like receptor 4 (TLR4) activation by lipopolysaccharide in Gram-negative bacteria) triggers nuclear factor Kappa B (NF-κB) signaling and induction of profibrotic mediators such as transforming growth factor-β (TGF-β), connective tissue growth factor (CTGF), and interleukin-6 (IL-6), which can sustain an inflammatory and fibrotic cascade that can drive tubular atrophy, capillary rarefaction, and irreversible nephron loss [11,12,13].

While the link between acute kidney injury (AKI) and CKD progression is well established, the long-term impact of UTI-derived insults in the absence of overt AKI is less clearly delineated [14,15]. Recurrent subclinical inflammatory stimuli may gradually shift the renal microenvironment toward maladaptive repair, manifesting as chronic interstitial nephritis, reduced glomerular filtration rate (GFR), or new-onset proteinuria. Furthermore, in patients with preexisting CKD, UTIs can accelerate disease progression, particularly when complicated by obstruction or urosepsis [16,17,18]. Cohort studies have shown that recurrent UTIs are associated with faster eGFR decline and increased risk of kidney failure [19]. In one large-scale analysis of CKD patients, repeat UTI episodes independently predicted mortality and renal function deterioration [18,20,21].

An additional challenge arises from the growing burden of antimicrobial resistance (AMR) in UTIs. The spread of extended-spectrum β-lactamase (ESBL)-producing *E. coli* and *Klebsiella* strains, as well as vancomycin-resistant *Enterococcus* (VRE), has led to higher rates of recurrence, treatment failure, and prolonged inflammatory exposure [22,23,24]. In CKD populations, reduced renal reserve, altered pharmacokinetics, and frequent antibiotic exposure create a vicious cycle of infection, inflammation, and nephrotoxicity [25,26]. Recognizing the contribution of specific uropathogens and their virulence or resistance patterns is essential for improving prevention, antimicrobial stewardship, and renal monitoring strategies.

In this review, we examine the evidence linking UTIs caused by *Escherichia coli*, *Klebsiella* spp., and *Enterococcus* spp. to CKD onset and progression, highlighting shared mechanisms, pathogen-specific pathways, and clinical implications.

## 2. Epidemiology of UTI and CKD Overlap

UTIs are among the most frequent bacterial infections across all age groups, with a lifetime incidence exceeding 50% in women and a substantial burden of recurrence, particularly in vulnerable populations [3,27]. Recurrent UTIs, commonly defined as ≥2 episodes within 6 months or ≥3 within 1 year, affect a significant proportion of patients and are associated with increased healthcare utilization, antibiotic exposure, and a higher risk of complications [27,28].

From an epidemiological perspective, the overlap between UTIs and CKD is increasingly recognized, particularly among older adults and individuals with metabolic comorbidities. CKD affects more than 10% of the global population and is projected to become one of the leading causes of mortality worldwide [29,30]. Patients with CKD exhibit a significantly increased susceptibility to UTIs due to multiple converging factors, including immune dysfunction, uremic toxin accumulation, impaired neutrophil and lymphocyte activity, and frequent exposure to healthcare-associated pathogens [20]. In addition, structural and functional abnormalities of the urinary tract—such as impaired urine concentration, reduced antimicrobial peptide activity, and urinary stasis—further predispose CKD patients to infection [31].

Conversely, UTIs—especially when recurrent or complicated—have been associated with an increased risk of CKD progression. Epidemiological and clinical data suggest that recurrent episodes of upper urinary tract infection (e.g., pyelonephritis) can lead to renal scarring, progressive nephron loss, and long-term decline in GFR [3,32,33].

This association was particularly pronounced in high-risk populations. Patients with diabetes mellitus, for example, exhibit both increased incidence and severity of UTIs and an accelerated trajectory of CKD progression, reflecting the combined effects of immune dysregulation, hyperglycemia-induced bacterial growth, and microvascular damage [34,35,36]. Elderly individuals also represent a key risk group, characterized by immunosenescence, higher rates of catheterization, and increased exposure to multidrug-resistant organisms, all of which contribute to recurrent UTIs and worsening renal function [28,37,38,39,40].

Healthcare-associated UTIs, particularly catheter-associated urinary tract infections (CAUTIs), further complicate this epidemiological landscape. These infections are strongly linked to biofilm-forming pathogens such as *Klebsiella* spp. and *Enterococcus* spp., which are associated with persistence, recurrence, and antimicrobial resistance [28,41,42].

Importantly, recent epidemiological insights emphasize that the burden of UTIs in CKD is not limited to acute episodes but includes subclinical or persistent infections that may contribute to chronic inflammation and progressive renal injury. This paradigm shift, from acute infection models to chronic, low-grade infection frameworks, is a key development in the field [28,41,43,44,45].

Taken together, these data support the concept that UTIs and CKD are interconnected conditions sharing common risk factors, overlapping patient populations, and mutually reinforcing pathophysiological mechanisms. Understanding this epidemiological interplay is essential for identifying high-risk patients and developing targeted preventive and therapeutic strategies.

## 3. Uropathogens of Interest: Microbiological and Virulence Profiles

Uropathogenic *Escherichia coli* (UPEC) remains the leading cause of urinary tract infections, accounting for the majority of both community-acquired and recurrent cases [46,47,48]. The pathogenic success of UPEC is largely driven by its ability to adhere to urothelial cells via type 1 and P fimbriae, enabling efficient colonization of the urinary tract [49,50]. Following adhesion, UPEC invades bladder epithelial cells and forms intracellular bacterial communities (IBCs), which promote immune evasion and persistence [51,52]. These bacteria can establish quiescent intracellular reservoirs that persist after treatment and serve as sources of recurrent infection [53,54]. UPEC also produces multiple virulence factors, including α-hemolysin and siderophores, which contribute to epithelial injury and inflammatory activation [55,56,57]. Ascending infection to the kidney may result in pyelonephritis, triggering inflammatory pathways that contribute to tubular damage and fibrosis [58,59].

*Klebsiella pneumoniae* is a significant uropathogen in complicated and healthcare-associated urinary tract infections, particularly among catheterized, hospitalized, and immunocompromised patients. Its clinical relevance is amplified by biofilm formation and frequent multiple drug resistance (MDR) [5,60,61,62]. A central virulence determinant of *K. pneumoniae* is the polysaccharide capsule, which contributes to immune evasion, while additional virulence-associated traits include fimbriae, siderophores, and factors that support survival on mucosal and abiotic surfaces. Recent reviews emphasize that the virulence profile varies between strains, helping explain differences in pathogenicity and clinical severity [63,64].

From a clinical perspective, *Klebsiella* UTIs are especially concerning when they involve ESBL-producing or carbapenem-resistant strains, because therapeutic options become narrower and treatment failure becomes more likely. This issue has also been highlighted in kidney-transplant and other high-risk populations [5,27,65]. For review of CKD angle, the key take-home message is that *Klebsiella* combines three properties directly relevant to the risk of chronic renal injury: persistence, device-associated biofilm formation, and antimicrobial resistance. Together, these traits increase the probability of prolonged infection, recurrent inflammatory injury, and delayed microbiological clearance [62,64,65].

*Enterococcus* spp. (*Enterococcus faecalis* and *Enterococcus faecium*) are well-recognized causes of healthcare-associated UTIs, particularly among patients with urinary catheters, prior antibiotic exposure, urinary tract abnormalities, transplantation, diabetes, or other clinical vulnerabilities [66,67]. A major reason enterococci are difficult uropathogens is that they combine intrinsic antimicrobial tolerance with marked genomic plasticity and an ability to acquire additional resistance determinants. Recent reviews continue to emphasize vancomycin resistance and broader last-resort antibiotic resistance as major clinical threats [66,68,69]. With respect to pathogenesis, enterococci express adhesion- and biofilm-associated factors that support colonization of host tissues and medical devices. Biofilm biology is particularly relevant in CAUTI settings because it promotes persistence and makes eradication more difficult [70].

The clinical importance of this persistence phenotype is that enterococcal UTIs are often not dominated by dramatic acute cytotoxicity, but by ongoing colonization, recurrence, and treatment difficulty. That pattern makes them particularly relevant to a CKD-focused review, where repeated low-grade inflammatory injury may matter as much as overt acute damage [66,67,70]. For the manuscript, the most defensible framing is that enterococci should be discussed as persistence-adapted uropathogens: they are clinically important not because they are always the most aggressive organisms, but because they are well-suited to survive, recur, and resist therapy in fragile hosts [66,68,69].

## 4. Pathophysiological Links Between UTIs and CKD

The relationship between UTIs and CKD is increasingly understood as a continuum driven by interconnected pathophysiological processes rather than isolated acute events. While most UTIs resolve without long-term consequences, recurrent or persistent infections may initiate sustained inflammatory and repair responses that progressively impair renal structure and function. This process is influenced not only by pathogen-specific virulence and persistence mechanisms but also by host susceptibility, immune activation, and maladaptive tissue remodeling.

### 4.1. Ascending Infection and Renal Involvement

The pathophysiological connection between UTIs and CKD begins with the ability of lower urinary tract infections to ascend toward the renal pelvis and parenchyma, resulting in acute pyelonephritis and direct tubulointerstitial injury. This mechanism is particularly relevant in recurrent or febrile UTIs, where kidney involvement may result in permanent structural sequelae rather than transient inflammation [20,71]. By contrast, uncomplicated lower UTIs without renal involvement are less consistently associated with permanent structural kidney damage or long-term decline in renal function.

Clinical and epidemiologic studies support the association between pyelonephritis and renal scarring, particularly in susceptible individuals, while recurrent upper urinary tract infections increase the likelihood of progressive nephron loss and long-term decline in renal function [72,73].

### 4.2. Inflammation and Innate Immune Activation

Once uropathogens reach the urothelium and renal tissue, innate immune pathways are activated through pattern-recognition receptors, cytokine signaling, and recruitment of inflammatory cells. Although these responses are essential for bacterial clearance, excessive or persistent activation may amplify tissue injury and promote chronic renal damage [74,75].

Inflammatory mediators such as IL-6 and IL-8 are consistently elevated during UTIs and correlate with upper urinary tract involvement and inflammatory burden. In parallel, CKD itself is characterized by chronic low-grade inflammation, in which persistent cytokine signaling contributes to endothelial dysfunction, tubular injury, and fibrotic remodeling [76,77]. Consequently, recurrent UTIs may contribute to CKD progression not only through bacterial persistence but also through prolonged host inflammatory responses [20,74].

### 4.3. Inflammasome Activation and Oxidative Stress

An additional mechanistic layer involves activation of the NLR family pyrin domain-containing 3 (NLRP3) inflammasome, which links infection-associated danger signals to caspase-1 activation and the release of IL-1β and IL-18, thereby amplifying renal inflammation [20,78,79]. Persistent inflammasome activation has been associated with maladaptive repair following acute kidney injury and may represent a biologically plausible mechanism contributing to chronic renal injury after recurrent pyelonephritic episodes. However, direct evidence specifically linking NLRP3 activation to UTI-associated CKD progression in humans remains limited [78,80].

At the same time, infection-driven inflammation promotes oxidative stress (OS), characterized by excessive reactive oxygen species (ROS) generation, mitochondrial dysfunction, and tubular epithelial injury [81,82]. Renal tubular epithelial cells are particularly vulnerable to oxidative damage, and unresolved inflammatory signaling may impair tissue recovery and favor progression toward chronic injury [81,83]. Although oxidative stress pathways are well established in experimental models of kidney injury, their precise contribution to long-term renal damage following recurrent UTIs remains incompletely characterized.

Together, inflammasome activation and OS may contribute to a self-sustaining cycle of inflammation and tissue injury that favors progressive renal dysfunction.

### 4.4. Fibrosis and Maladaptive Repair

Fibrosis represents the final common pathway of progressive CKD, with transforming growth factor-β (TGF-β)/Smad signaling playing a central role in extracellular matrix deposition and tubulointerstitial remodeling [84,85]. Following recurrent or severe infection, injured tubular epithelial cells may adopt maladaptive phenotypes that perpetuate profibrotic signaling and myofibroblast activation rather than complete tissue repair [85,86].

Accordingly, recurrent UTIs may induce a persistent profibrotic microenvironment even after microbiological clearance, potentially contributing to interstitial fibrosis, nephron loss, and progressive decline in renal function.

Importantly, much of the current understanding regarding maladaptive repair and profibrotic signaling derives from the broader AKI-to-CKD and chronic fibrosis literature rather than from studies specifically focused on recurrent UTIs.

### 4.5. Integration: From Acute Infection to Chronic Kidney Disease

Taken together, recurrent or severe UTIs may be conceptually linked to chronic renal injury through interconnected pathways involving inflammation, oxidative stress, and maladaptive repair. Rather than representing isolated acute events, recurrent or persistent UTIs may therefore contribute to cumulative renal damage over time [86,87].

In this framework, the balance between effective bacterial clearance and persistent inflammation may influence whether renal tissue recovers or progresses toward chronic interstitial fibrosis and nephron loss. This integrated pathophysiological framework is consistent with current concepts of AKI-to-CKD transition and chronic inflammatory kidney injury [87,88].

Nevertheless, many of these mechanisms have been primarily characterized in experimental or in vitro models, whereas direct clinical evidence linking recurrent UTIs to progressive CKD in humans remains comparatively limited. Furthermore, the relative contribution of infection itself versus underlying host susceptibility factors remains incompletely understood.

Importantly, much of the current clinical evidence linking recurrent UTIs to CKD progression is derived from retrospective or observational studies, while prospective longitudinal data remain relatively limited.

**Figure 1:**
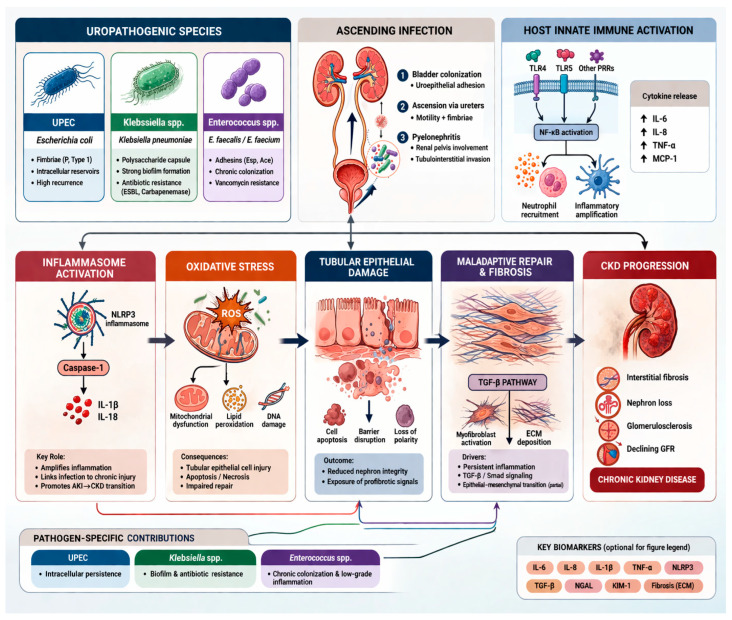
Integrated mechanistic model of pathogen-specific and host-mediated pathways driving UTI-associated CKD (Figure created in Canva, https://www.canva.com). The figure summarizes the major mechanisms involved in UTI-associated CKD progression, including ascending infection, inflammatory activation, oxidative stress, tubular injury, and fibrosis. Pathogen-specific persistence strategies of UPEC, *Klebsiella* spp., and *Enterococcus* spp. are integrated with representative biomarker profiles and downstream pathways of renal injury. Abbreviations: UTI—Urinary tract infection, CKD—Chronic kidney disease, UPEC—Uropathogenic, *Escherichia coli*, TLR—Toll-like receptor, NLRP3—NLR family pyrin domain-containing 3, NF-κB—Nuclear factor kappa B, IL—Interleukin, TNF-α—Tumor necrosis factor alpha, MCP-1 (CCL2)—Monocyte chemoattractant protein-1, ROS—Reactive oxygen species, OS—Oxidative stress, NGAL—Neutrophil gelatinase-associated lipocalin, KIM-1—Kidney injury molecule-1, TGF-β—Transforming growth factor beta, ECM—Extracellular matrix, 8-OHdG—8-hydroxy-2′-deoxyguanosine, AKI—Acute kidney injury, ESBL—Extended-spectrum β-lactamase, CRP—C-reactive protein.

The complex interplay of pathogen-specific mechanisms and host-mediated responses described above can be integrated into a unified model of renal injury. A schematic overview of these interconnected mechanisms is presented in Figure 1.

## 5. Comparative Role of Uropathogens in CKD Progression

Although UTIs are frequently approached as a relatively homogeneous clinical entity, accumulating evidence indicates that the long-term renal consequences of infection depend substantially on pathogen-specific virulence and persistence strategies. In particular, UPEC, *Klebsiella* spp., and *Enterococcus* spp. exhibit distinct mechanisms of host interaction, immune evasion, and chronic persistence, which may differentially influence mechanisms potentially associated with CKD progression.

UPEC remains the prototypical uropathogen and is uniquely adapted to establish recurrent infections through intracellular persistence. Following adhesion to urothelial cells via type 1 fimbriae, UPEC invade host cells, forming intracellular bacterial communities and quiescent intracellular reservoirs. These reservoirs enable evasion from both immune surveillance and antibiotic therapy, thereby facilitating recurrence. Repeated infection-resolution cycles may contribute to cumulative inflammatory injury and progressive tubular damage [3,71].

In contrast, *Klebsiella pneumoniae* and related species rely predominantly on extracellular persistence mechanisms, particularly biofilm formation and antimicrobial resistance. Their ability to generate structured biofilms on urinary catheters and epithelial surfaces enhances bacterial survival while limiting antibiotic penetration and host immune clearance. Moreover, the growing prevalence of ESBL and carbapenem-resistant strains further complicates eradication and favors prolonged infection. In susceptible patients, especially those with diabetes, recurrent healthcare exposure, or pre-existing CKD, these mechanisms may contribute to sustained renal inflammation and delayed recovery [5,63]. However, direct evidence linking these microbiological features to specific long-term renal phenotypes remains limited.

**Figure 2:**
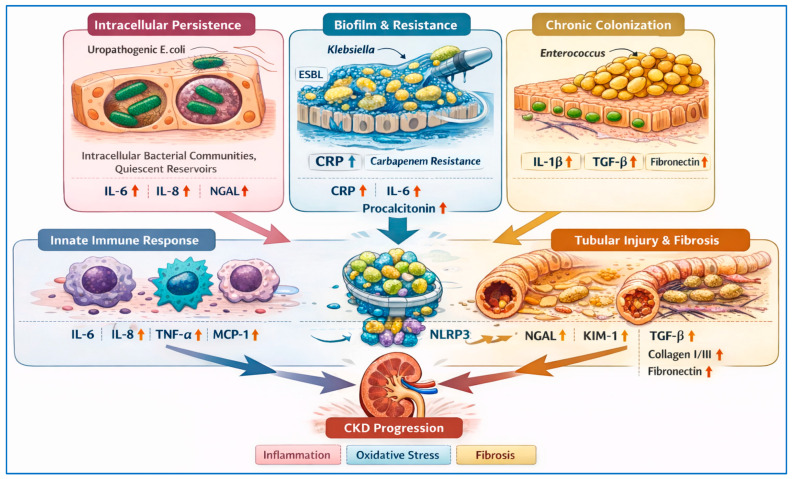
Integrated pathogen-specific and host-mediated mechanisms linking urinary tract infections (UTI) to chronic kidney disease progression: a hybrid model incorporating persistence strategies and biomarker profiles (Figure created in Canva, https://www.canva.com). The figure summarizes the distinct persistence strategies of major uropathogens and their convergence toward shared pathways of renal injury. UPEC is characterized by intracellular persistence and recurrent infection; *Klebsiella* spp. by biofilm formation and antimicrobial resistance; and *Enterococcus* spp. by chronic colonization and low-grade inflammation. These pathogen-specific mechanisms are integrated with representative biomarker profiles reflecting inflammation, tubular injury, and fibrotic remodeling. The model highlights how microbial persistence and host-mediated responses may collectively contribute to CKD progression. Abbreviations: UTI, urinary tract infection; CKD, chronic kidney disease; UPEC, uropathogenic *Escherichia coli*; IL, interleukin; NGAL, neutrophil gelatinase-associated lipocalin; CRP, C-reactive protein; TGF-β, transforming growth factor beta; TNF-α, tumor necrosis factor alpha; MCP-1, monocyte chemoattractant protein-1; KIM-1, kidney injury molecule-1; ECM, extracellular matrix; ESBL, extended-spectrum β-lactamase.

*Enterococcus* spp. represents a distinct pathogenic paradigm characterized more by persistence and immune tolerance than by aggressive virulence. These organisms are intrinsically resistant to several antimicrobial classes and are frequently associated with chronic urinary colonization, particularly in catheterized or hospitalized individuals. Unlike UPEC, enterococcal infections are often less acutely destructive but may favor persistent low-grade inflammation that could contribute to chronic renal injury over time. This chronic inflammatory profile aligns closely with the broader pathophysiological mechanisms illustrated in Figure 2, where persistent immune activation drives maladaptive repair and fibrotic remodeling. In addition to the described pathways, key biomarkers associated with each stage of disease progression are indicated, including inflammatory cytokines (IL-6, IL-8, TNF-α, MCP-1), inflammasome-related mediators (IL-1β, IL-18), oxidative stress markers (ROS, 8-OHdG), tubular injury markers (NGAL, KIM-1), and fibrosis-associated proteins (TGF-β, collagen, fibronectin). These biomarkers provide a translational link between molecular mechanisms and clinical assessment of kidney injury [66,88,89].

Despite these differences, these pathogens appear to converge on shared downstream pathways of kidney injury, including chronic inflammation, tubular damage, oxidative stress, and fibrotic remodeling. However, they differ substantially in how these mechanisms are initiated and sustained. Although these pathogens exhibit distinct microbiological persistence strategies, their downstream effects may involve overlapping inflammatory and injury-related pathways. UPEC is primarily linked to recurrent intracellular persistence and repeated inflammatory episodes, *Klebsiella* spp. to biofilm-associated persistence and antimicrobial resistance, whereas *Enterococcus* spp. is more commonly associated with chronic colonization and sustained low-grade immune activation. However, these proposed differences should be interpreted as conceptual mechanistic frameworks rather than established pathogen-specific models of CKD progression.

Importantly, pathogen-specific mechanisms such as intracellular persistence and biofilm formation are well established in experimental settings; however, their precise contribution to long-term renal outcomes in clinical populations remains difficult to quantify.

This pathogen-specific heterogeneity suggests that the renal consequences of UTIs are unlikely to be uniform across all infections. Instead, CKD risk appears to depend on the interaction between microbial persistence strategies, host susceptibility, and the efficiency of inflammatory resolution. As illustrated in Figure 2, incomplete pathogen clearance and persistent inflammation may favor maladaptive renal repair and progressive fibrosis. However, current evidence remains insufficient to definitively link individual uropathogens with distinct renal fibrosis patterns, biomarker signatures, or long-term CKD phenotypes in human populations. These observations support the need for pathogen-oriented diagnostic and therapeutic approaches, particularly in high-risk populations such as patients with diabetes, recurrent UTIs, urinary catheterization, or pre-existing CKD.

However, the relative contribution of individual pathogens to long-term renal outcomes remains difficult to isolate in clinical settings because recurrent UTIs frequently coexist with major CKD risk factors, including diabetes, urinary tract abnormalities, catheterization, and advanced age.

Importantly, interpretation of long-term renal outcomes remains complicated by major confounding factors. Conditions such as diabetes mellitus, urinary catheterization, advanced age, immune dysfunction, and pre-existing CKD may independently predispose patients to both recurrent UTIs and progressive renal decline.

The pathogen-specific and host-mediated mechanisms involved in UTI-associated CKD progression are summarized in Figure 2. The schematic highlights the distinct persistence strategies of UPEC, *Klebsiella* spp., and *Enterococcus* spp., as well as their convergence on common pathways of renal injury, including inflammation, oxidative stress, tubular dysfunction, and fibrosis.

## 6. Antibiotic Resistance and Its Impact on CKD

Antibiotic resistance represents a critical factor in the transition from acute UTI to persistent or recurrent disease, thereby increasing the risk of chronic kidney injury. Infections caused by multidrug-resistant uropathogens are more difficult to eradicate, often requiring prolonged or repeated courses of antibiotics, which may delay bacterial clearance and sustain inflammatory responses within the kidney. This is particularly relevant in complicated UTIs, where persistent infection can lead to recurrent episodes of pyelonephritis and cumulative tubulointerstitial damage [5,27].

A major clinical consequence of antimicrobial resistance is treatment failure or suboptimal therapy, thereby allowing bacteria to persist in the urinary tract. In this context, pathogens such as ESBL-producing *Klebsiella pneumoniae* or VRE are associated with prolonged infection duration and increased risk of recurrence. Persistent infection leads to sustained activation of inflammatory pathways, including NF-κB signaling and cytokine production, thereby amplifying renal injury and promoting progression toward CKD [63,66].

In addition to pathogen persistence, antibiotic therapy itself may contribute to renal injury through nephrotoxicity. Aminoglycosides, for example, are well known to induce acute tubular injury through accumulation in proximal tubular epithelial cells, leading to OS, mitochondrial dysfunction, and cell death. Although often necessary in severe infections, repeated or prolonged exposure to nephrotoxic agents may increase the risk of long-term renal impairment, particularly in patients with pre-existing kidney disease or recurrent complicated UTIs [90,91,92,93,94].

Another emerging aspect is the impact of antibiotics on the urinary and gut microbiome. Broad-spectrum antibiotic use can disrupt microbial homeostasis, leading to dysbiosis that favors colonization by resistant or opportunistic pathogens. This altered microbial environment may increase susceptibility to recurrent UTIs and contribute indirectly to chronic inflammation. In turn, microbiome disruption has been linked to systemic immune dysregulation and may influence CKD progression through inflammatory and metabolic pathways [95,96,97,98].

From a pathophysiological perspective, antibiotic resistance and its consequences integrate directly into the mechanisms illustrated in Figure 2. Persistent infection due to ineffective therapy sustains inflammatory signaling, promotes OS, and drives maladaptive repair processes, ultimately leading to fibrosis and nephron loss. Thus, antimicrobial resistance should not be viewed solely as a microbiological problem but as a key contributor to the risk of CKD in patients with recurrent or complicated UTIs.

## 7. Host Factors Modulating the UTI–CKD Axis

The progression from UTI to CKD is not determined solely by pathogen characteristics, but is strongly influenced by host-related factors that modulate susceptibility, immune response, and repair mechanisms. Among these, metabolic conditions, immune status, and microbiome composition play a central role in determining whether an infection resolves or progresses to chronic renal injury.

One of the most important host-related risk factors is diabetes mellitus, which is consistently associated with both increased susceptibility to UTIs and accelerated CKD progression. Hyperglycemia promotes bacterial growth in the urinary tract, impairs neutrophil function, and disrupts innate immune responses. In addition, diabetic patients exhibit microvascular damage, oxidative stress, and baseline inflammation, all of which amplify renal vulnerability to infection-induced injury. As a result, UTIs in diabetic individuals are more likely to be severe, recurrent, and associated with long-term renal consequences [36,99,100,101].

Beyond diabetes, altered immune function represents a key determinant of the UTI–CKD axis. Both immunosuppressed patients (e.g., transplant recipients) and elderly individuals exhibit impaired pathogen clearance, leading to prolonged infection and increased risk of persistence. Dysregulation of innate and adaptive immune responses may result in either insufficient bacterial elimination or excessive inflammatory activation, both of which contribute to tissue injury. This imbalance is particularly relevant in recurrent infections, where repeated immune activation promotes chronic inflammation and fibrotic remodeling [5,66,101,102,103].

Another emerging factor is the role of the urinary and gut microbiome in modulating susceptibility to infection and inflammation. Disruption of microbial homeostasis, often due to antibiotic exposure or underlying disease, can lead to dysbiosis that favors colonization by uropathogens. In addition, the gut–kidney axis has been increasingly recognized as a contributor to systemic inflammation, with microbial metabolites influencing immune responses and renal function. Alterations in the microbiome may therefore not only predispose to recurrent UTIs but also contribute to CKD progression through inflammatory and metabolic pathways [104,105,106,107,108].

Host structural and functional abnormalities of the urinary tract also play a significant role. Conditions such as urinary obstruction, vesicoureteral reflux, and catheterization facilitate bacterial ascent and persistence, increasing the likelihood of renal involvement. These factors are particularly important in recurrent or complicated UTIs, where mechanical disruption of normal urinary flow promotes sustained infection and repeated renal injury [109,110].

Importantly, these host factors interact with pathogen-specific mechanisms; for example, intracellular persistence of UPEC is more likely to result in recurrence in immunocompromised hosts, while biofilm-associated infections by *Klebsiella* spp. are particularly problematic in catheterized patients. Similarly, chronic colonization by *Enterococcus* spp. is favored in dysbiotic environments with impaired immune surveillance.

Taken together, these observations support a multifactorial model in which host susceptibility determines the trajectory of infection. Rather than acting as isolated risk factors, metabolic, immunological, and structural conditions collectively shape the balance between bacterial clearance and persistence. This, in turn, influences whether UTIs remain acute and self-limited or evolve into chronic processes that contribute to CKD progression.

### Microbiome Dysbiosis and the Gut–Kidney–Urinary Axis

Emerging evidence suggests that microbiome alterations may represent an additional mechanistic link between recurrent UTIs and CKD progression [111,112,113,114,115,116,117]. Both the intestinal and urinary microbiota contribute to immune regulation, epithelial barrier integrity, and colonization resistance against uropathogens. Disruption of these microbial communities, particularly following recurrent antibiotic exposure, may favor persistent colonization, recurrent infection, and chronic inflammatory activation.

In CKD, gut dysbiosis has been associated with increased intestinal permeability, systemic inflammation, oxidative stress, and accumulation of uremic toxins, all of which may further amplify renal injury [112,113,114,115,116]. At the same time, recurrent UTIs and repeated antimicrobial therapy may alter urinary microbial diversity and impair protective commensal populations, potentially facilitating pathogen persistence and recurrent infection [117].

Although the precise contribution of microbiome alterations to the UTI–CKD axis remains incompletely understood, growing evidence supports a bidirectional interaction between microbial dysbiosis, host immune responses, and chronic renal inflammation [111]. Further translational and longitudinal studies are needed to clarify whether microbiome-targeted interventions can reduce recurrent infections and limit CKD progression.

## 8. Biomarkers Linking UTI to CKD Progression

The identification of reliable biomarkers linking UTIs to CKD progression is important for early diagnosis, risk stratification, and monitoring of renal injury. Because infection-associated renal damage involves overlapping inflammatory, tubular injury, oxidative stress, and fibrotic pathways, no single marker is likely to capture the full trajectory of disease. A multimarker approach may therefore provide a more informative assessment of patients with recurrent or complicated UTIs.

Among tubular injury biomarkers, neutrophil gelatinase-associated lipocalin (NGAL) is one of the most extensively studied. NGAL is rapidly released by injured tubular epithelial cells and neutrophils in response to inflammation and epithelial stress. Elevated NGAL levels have been associated with AKI and early CKD-related changes, and in the context of upper urinary tract involvement, they may indicate subclinical tubular damage before major changes in GFR become evident [118,119,120,121].

Systemic inflammatory biomarkers, particularly CRP, may help assess infection severity and systemic inflammatory burden. Elevated CRP levels are commonly observed in complicated UTIs and pyelonephritis and may also reflect persistent inflammation, a recognized contributor to CKD progression through endothelial dysfunction and fibrotic signaling [122,123].

Cytokine-based markers provide additional information on immune activation. Increased levels of IL-6, IL-8, TNF-α, and MCP-1 have been reported in UTI and are associated with inflammatory burden, bacterial load, and tissue injury. These mediators are also involved in CKD pathophysiology, supporting their inclusion in biomarker panels to identify patients at increased risk of long-term renal damage [66,124,125,126,127].

Beyond classical inflammatory and injury markers, neuroimmune mediators are emerging as potential contributors to UTI pathophysiology. Neuropeptides and neurotransmitter-related pathways, including substance P and catecholamine signaling, may influence epithelial barrier function, immune responses, and bladder inflammation. Although this area remains investigational, such mediators could help connect local infection, chronic inflammation, and systemic renal outcomes [28,74,128,129].

Importantly, these biomarkers should be interpreted as complementary indicators rather than isolated diagnostic tools. Inflammatory cytokines reflect innate immune activation, IL-1β and IL-18 correspond to inflammasome signaling, NGAL and KIM-1 indicate tubular injury, and TGF-β and extracellular matrix proteins reflect fibrotic remodeling. This integrated interpretation supports the translational value of combining mechanistic pathways with measurable clinical parameters.

An integrated overview of pathogen-specific mechanisms and associated biomarker profiles is presented in Table 1, highlighting how different uropathogens contribute to renal injury through distinct yet converging pathways. As shown in Table 1, pathogen-specific biomarker patterns may help link microbial persistence mechanisms to clinical monitoring and therapeutic decision-making.

## 9. Clinical Implications

The recognition that recurrent or persistent UTIs may contribute to CKD progression has important therapeutic and clinical implications. Beyond the treatment of acute infection, management strategies should increasingly consider the potential impact of recurrent infection on long-term renal health, while aiming to limit chronic inflammation and identify patients at increased risk of kidney injury.

Early and appropriate antimicrobial therapy remains essential, particularly in febrile or complicated UTIs associated with upper urinary tract involvement. However, the growing prevalence of antimicrobial resistance—especially among *Klebsiella* spp. and *Enterococcus* spp.—has complicated treatment selection and may increase the likelihood of persistent infection and sustained inflammatory responses [5,63]. In this context, antimicrobial stewardship and pathogen-guided therapy may be important not only for infection control but also for preservation of renal function.

For recurrent UPEC infections, intracellular bacterial persistence may partially explain recurrent infection and reduced therapeutic efficacy. This observation underscores the need for therapeutic approaches targeting intracellular reservoirs to reduce the risk of recurrence. In contrast, biofilm-associated infections caused by *Klebsiella* spp. may require strategies to disrupt biofilm architecture and enhance antimicrobial penetration, particularly in catheter-associated UTIs.

Risk stratification is also clinically relevant. Patients with diabetes, urinary tract abnormalities, indwelling catheters, recurrent UTIs, or pre-existing CKD may be particularly susceptible to infection-associated renal impairment. Importantly, these conditions may act not only as susceptibility factors for recurrent infection but also as independent drivers of CKD progression, complicating the interpretation of infection-related renal outcomes. In these populations, closer renal monitoring and earlier intervention may help reduce cumulative kidney injury.

Biomarker-guided approaches may further improve clinical management by enabling earlier identification of tubular injury and persistent inflammatory activity before a substantial decline in renal function becomes evident. Integration of inflammatory, tubular injury, and fibrosis-associated biomarkers into clinical practice could eventually support individualized monitoring and therapeutic decision-making.

Emerging therapeutic strategies are increasingly directed toward mechanisms of persistence and chronic inflammation rather than bacterial eradication alone. Approaches targeting biofilm formation, inflammasome signaling, oxidative stress, and maladaptive fibrotic repair are currently under investigation and could become increasingly relevant in recurrent or complicated UTIs associated with CKD progression.

Taken together, these observations support a broader clinical perspective in which UTIs should be considered not only acute infectious events, but also potential modifiers of long-term renal outcomes in susceptible individuals. This concept reinforces the importance of early recognition, pathogen-specific management, and long-term renal monitoring in high-risk patient populations.

Current clinical guidelines also support the importance of risk stratification and individualized management in patients with recurrent or complicated UTIs. The European Association of Urology (EAU) Guidelines on Urological Infections emphasize the role of antimicrobial stewardship, catheter management, and targeted therapy in reducing recurrent infection and limiting antimicrobial resistance [130]. Similarly, Kidney Disease: Improving Global Outcomes (KDIGO) recommendations highlight the importance of early identification and monitoring of patients at increased risk of CKD progression, particularly those with recurrent infections, diabetes, or pre-existing renal dysfunction [131]. Although current guidelines do not yet fully integrate mechanistic biomarkers or microbiome-based approaches, these strategies may become increasingly relevant in future personalized management frameworks.

## 10. Future Directions

Despite significant advances in understanding the pathophysiological links between UTIs and CKD, several key areas remain to be explored in order to improve prevention, diagnosis, and treatment strategies. Future research should focus on targeting pathogen persistence, refining biomarker-based approaches, and developing personalized interventions that integrate microbial and host factors.

One of the most promising directions involves the development of **anti-biofilm therapies**, particularly for pathogens such as *Klebsiella* spp., where biofilm formation plays a central role in persistence and antibiotic resistance. Novel strategies—including biofilm-disrupting approaches, quorum-sensing-targeted interventions, and bacteriophage therapy—are emerging as promising alternatives or adjuncts for chronic and catheter-associated UTIs, although stronger clinical validation remains needed [132,133,134,135,136].

Another important area is the development of **vaccines against uropathogens**, especially UPEC, which remains the leading cause of recurrent UTIs. Several vaccine-based strategies for recurrent urinary tract infections, particularly those targeting UPEC, are currently under investigation [137,138,139]. Successful vaccination strategies could reduce recurrence rates and, consequently, limit recurrent renal injury, representing a major step forward in preventing CKD progression.

The **modulation of the microbiome** represents an emerging and highly relevant field. Advances in understanding the gut–kidney and urinary microbiome axes suggest that restoring microbial balance through probiotics, prebiotics, or microbiota-targeted therapies may reduce susceptibility to infection and dampen chronic inflammation. This approach may be particularly valuable in patients with recurrent UTIs and dysbiosis driven by repeated antibiotic exposure.

In parallel, there is increasing interest in developing **predictive biomarker panels** that identify patients at high risk of CKD progression following UTIs. Rather than relying on a single marker, future approaches will likely integrate multiple biomarkers—including inflammatory cytokines, tubular injury markers, and fibrosis-related proteins—alongside clinical and microbiological data. The incorporation of machine learning and precision medicine frameworks may further enhance risk prediction and guide individualized management strategies.

Finally, innovative research directions are exploring the role of **neuroimmune interactions** in UTI pathophysiology. The interplay between the nervous system and immune responses in the urinary tract may influence both susceptibility to infection and the transition to chronic inflammation. Although still in early stages, this field may open new therapeutic avenues targeting neural signaling pathways to modulate inflammation and prevent long-term renal damage.

Overall, future research should move toward an integrated, systems-level understanding of the UTI–CKD axis, combining pathogen biology, host responses, and clinical data. Such an approach will be essential for developing effective strategies to prevent the progression from acute infection to chronic kidney disease.

### Quality of Evidence, Limitations, and Translational Challenges

Although growing evidence supports biologically plausible links between recurrent UTIs and CKD progression, the quality and strength of the available evidence remain heterogeneous. Several mechanisms discussed in this review, including intracellular persistence, inflammasome activation, oxidative stress, and biofilm-associated injury, are strongly supported by experimental and in vitro studies. However, their precise contribution to long-term renal outcomes in human populations remains incompletely established.

Much of the current clinical evidence linking recurrent UTIs to CKD progression is derived from observational and retrospective studies, whereas prospective longitudinal investigations remain relatively limited. Consequently, definitive causal relationships are difficult to establish, particularly given the multifactorial nature of CKD and the influence of major confounding factors such as diabetes mellitus, advanced age, urinary catheterization, immune dysfunction, and pre-existing kidney disease.

In addition, important translational differences exist between experimental models and clinical disease. Mechanisms that are reproducibly demonstrated in vitro or in animal models may not fully reflect the complexity and heterogeneity of human infections, particularly in patients with multiple comorbidities or recurrent healthcare exposure.

Therefore, while current evidence supports a biologically plausible association between recurrent UTIs and progressive renal injury, further longitudinal clinical studies and translational investigations are needed to better define causality, identify high-risk patient populations, and validate biomarker-guided therapeutic strategies.

## 11. Conclusions

UTIs are traditionally regarded as acute and self-limited conditions; however, accumulating evidence supports their potential role in CKD progression. This review highlights that the transition from infection to chronic renal injury likely involves a complex interplay between pathogen-specific persistence mechanisms, host susceptibility factors, and maladaptive repair processes. Distinct uropathogens, including UPEC, *Klebsiella* spp., and *Enterococcus* spp., employ different strategies to sustain infection, such as intracellular persistence, biofilm formation, and chronic colonization. Despite these differences, their pathogenic effects appear to converge on common downstream pathways involving inflammation, inflammasome activation, oxidative stress, and fibrosis. As summarized in Table 1 and Figure 1 and Figure 2, these mechanisms may contribute to tubular injury, nephron loss, and progressive decline in renal function. Host-related factors also likely play a central role in determining disease trajectory. Conditions such as diabetes, immune dysregulation, advanced age, catheterization, and pre-existing kidney disease may increase susceptibility to infection while favoring persistent inflammatory and profibrotic responses. In parallel, antimicrobial resistance and treatment-related factors may further contribute to persistent infection and cumulative renal injury. The integration of biomarker-based approaches represents a promising strategy for early detection and risk stratification. Markers of inflammation, tubular injury, and fibrosis provide a translational bridge between molecular mechanisms and clinical practice, potentially enabling earlier identification of patients at increased risk of long-term renal impairment. Taken together, these findings support a broader clinical perspective in which recurrent or persistent UTIs should not be viewed solely as isolated infectious episodes, but also as potential contributors to chronic kidney injury in susceptible individuals. Early recognition, pathogen-specific management, and biomarker-guided monitoring may help reduce the risk of irreversible renal damage and improve patient outcomes. Future research should focus on improving diagnostic precision, validating biomarker-guided strategies, and developing targeted therapeutic approaches for the UTI–CKD axis. Current evidence appears strongest in the context of recurrent pyelonephritis and complicated UTIs involving the upper urinary tract, whereas the contribution of uncomplicated lower UTIs to CKD progression remains less clearly defined.

## Figures and Tables

**Table 1:** Integrated pathogen-specific mechanisms, biomarker profiles, and therapeutic implications in UTI-associated CKD progression.

Pathogen	Key Mechanism of Persistence	Dominant Pathophysiology	Inflammatory Biomarkers	Tubular Injury Markers	Fibrosis Markers	Clinical Interpretation	CKD Progression Pattern	Therapeutic Implications
**UPEC** (*E. coli*)	Intracellular bacterial communities, quiescent reservoirs	Recurrent acute inflammation and epithelial injury	IL-6, IL-8, TNF-α, MCP-1	NGAL, KIM-1	(secondary) TGF-β ↑	Recurrent UTIs, intracellular persistence	Stepwise cumulative damage → nephron loss	Target intracellular reservoirs; prolonged/targeted antibiotics; anti-adhesion strategies (e.g., FimH inhibitors)
***Klebsiella*** **spp.**	Biofilm formation + antimicrobial resistance (ESBL, carbapenemases)	Persistent infection with sustained inflammation	CRP, IL-6, Procalcitonin	NGAL ↑ (secondary)	TGF-β ↑ (chronic cases)	Severe/complicated UTI, treatment resistance	Chronic inflammation → fibrosis	Anti-biofilm strategies; guided antibiotic therapy (based on resistance); catheter management/removal
***Enterococcus*** **spp.**	Chronic colonization + intrinsic resistance	Low-grade persistent inflammation	IL-1β, MCP-1	Mild/ gradual injury	TGF-β, Fibronectin, Collagen I/III	Chronic colonization, subclinical inflammation	Progressive fibrosis → CKD	Long-term suppression strategies; microbiome modulation; cautious antibiotic use (avoid overtreatment)
**(Shared** **pathways)**	—	Inflammasome activation, oxidative stress	IL-1β, IL-18	NGAL, KIM-1	TGF-β, ECM proteins	Reflects a mechanistic cascade	Common final pathway: fibrosis	Anti-inflammatory therapies; antioxidant approaches; antifibrotic strategies (targeting TGF-β)

This table outlines the main uropathogens’ persistence mechanisms, pathophysiological pathways, and biomarker profiles in UTI, with a focus on CKD progression. UPEC involves intracellular persistence and epithelial injury; *Klebsiella* spp. relates to biofilm and resistance; *Enterococcus* spp. involves colonization and inflammation. These processes are reflected in specific biomarkers, yet all pathways converge on inflammasome activation, oxidative stress, and fibrosis, leading to nephron loss and CKD. The table also suggests therapeutic strategies targeting persistence, inflammation, and fibrosis, integrating microbiological, molecular, and clinical data. UTI: Urinary tract infection, CKD: Chronic kidney disease, UPEC: Uropathogenic *Escherichia coli*, IL: Interleukin, TNF-α: Tumor necrosis factor alpha, MCP-1: Monocyte chemoattractant protein-1, CRP: C-reactive protein, NGAL: Neutrophil gelatinase-associated lipocalin, KIM-1: Kidney injury molecule-1, TGF-β: Transforming growth factor beta, ECM: Extracellular matrix, ESBL: Extended-spectrum β-lactamase, ↑: Increased levels.

## Data Availability

No new data were created or analyzed in this study. Data sharing is not applicable to this article.
